# Subcutaneous daratumumab in Chinese patients with relapsed or refractory multiple myeloma: an open-label, multicenter, phase 1 study (MMY1010)

**DOI:** 10.1097/BS9.0000000000000193

**Published:** 2024-05-31

**Authors:** Gang An, Zheng Ge, Hongmei Jing, Jing Liu, Guoping Yang, Ru Feng, Zhongyuan Xu, Ming Qi, Jianping Wang, Juanjuan Song, Wei Zhou, Binbin Sun, Dian Zhu, Xi Chen, Canchan Cui, Lugui Qiu

**Affiliations:** a National Clinical Research Center for Hematological Disorders, State Key Laboratory of Experimental Hematology, Institute of Hematology and Blood Diseases Hospital, Chinese Academy of Medical Sciences and Peking Union Medical College, Tianjin 300020, China; b Department of Hematology, Zhongda Hospital Southeast University, Nanjing 210009, China; c Peking University Third Hospital, Beijing 100191, China; d Department of Hematology, The Third Xiangya Hospital of Central South University, Changsha 410013, China; e Department of Hematology, Nanfang Hospital of Southern Medical University, Guangzhou 510515, China; f Phase 1 Clinical Trial Department, Nanfang Hospital of Southern Medical University, Guangzhou 510515, China; g Janssen Research & Development, LLC, Spring House, PA 19477, USA; h Janssen Research & Development, LLC, Beijing 100025, China; i Janssen Research & Development, LLC, Shanghai 200231, China

**Keywords:** Chinese, Daratumumab, Multiple myeloma, Pharmacokinetic, Relapse/refractory, Safety, Subcutaneous

## Abstract

Despite recent progress in multiple myeloma (MM) treatments, most patients will relapse and require additional treatment. Intravenous daratumumab, a human IgGκ monoclonal antibody targeting CD38, has shown good efficacy in the treatment of MM. A subcutaneous version of daratumumab was formulated to reduce the burden of intravenous infusions. We aimed to investigate the efficacy and safety of subcutaneous daratumumab in Chinese patients with relapsed/refractory MM based on the demonstrated noninferiority of subcutaneous daratumumab to intravenous daratumumab, with a shorter administration time and reduced infusion-related reaction rate in global studies. This phase 1, multicenter study (MMY1010; ClinicalTrials.gov Identifier: NCT04121260) evaluated subcutaneous daratumumab in Chinese patients with relapsed/refractory MM after 1 prior line (n = 1) or ≥2 prior lines (n = 20) of therapy, including a proteasome inhibitor and an immunomodulatory drug. Primary endpoints were pharmacokinetics and safety. Mean (standard deviation) maximum trough concentration of daratumumab was 826 (335) μg/mL, which was consistent with prior studies of subcutaneous daratumumab and intravenous daratumumab. Safety was consistent with safety profiles observed in other daratumumab studies, with no new safety concerns identified. Incidences of infusion-related reactions and injection-site reactions were low and consistent with other subcutaneous daratumumab studies. At a median follow-up of 7.5 months, the overall response rate was 57.1%, with a very good partial response or better rate of 38.1% and complete response or better rate of 19.0%. Our results demonstrate a favorable benefit/risk profile of subcutaneous daratumumab in Chinese patients with relapsed/refractory MM, potentially impacting clinical administration of daratumumab in this population.

## 1. INTRODUCTION

In the United States, an estimated 34,470 people will be diagnosed with multiple myeloma (MM) in 2022 and approximately 12,640 patients will die from the disease.^1^ Based on a national population–based analysis (2012–2016), the standardized prevalence and incidence of MM in China’s mainland were 5.68 per 100,000 population and 1.15 per 100,000 person-years, respectively.^2^ Novel treatments, including proteasome inhibitors (PIs) and immunomodulatory drugs (IMiDs), have improved outcomes in MM^3^; however, most patients relapse and require additional treatment. Prognosis is dismal in patients who are heavily pre-treated or refractory to both PI and IMiD, with a median overall survival (OS) of only 8 to 9 months.^4,5^

Daratumumab (DARA) is a human IgGκ monoclonal antibody targeting CD38 with a direct on-tumor^6–9^ and immunomodulatory^10–12^ mechanism of action. Intravenous (IV) DARA has demonstrated efficacy across all lines of therapy, leading to its approval in many countries worldwide as monotherapy and in combination with standard-of-care regimens for the treatment of MM.^13,14^ In China, DARA was first approved in 2019 and is now indicated as monotherapy for patients with relapsed or refractory MM (RRMM) whose prior therapy included PI and IMiD in combination with lenalidomide and dexamethasone or bortezomib and dexamethasone for patients with MM who received ≥1 prior therapy and in combination with lenalidomide and dexamethasone or bortezomib, melphalan, and prednisone for patients with newly diagnosed MM who are ineligible for autologous stem cell transplant. In clinical studies, median duration of the first, second, and subsequent DARA IV infusions was 7, 4, and 3 hours, respectively.^14^ Approximately 40% of patients experienced infusion-related reactions (IRRs), which were manageable and occurred primarily during the first infusion.^14^

A subcutaneous (SC) formulation of DARA (DARA SC; DARA 1800 mg co-formulated with recombinant human hyaluronidase PH20 [rHuPH20; 2000 U/mL; ENHANZE^®^ drug delivery technology; Halozyme, Inc., San Diego, California]) was developed to reduce the burden of IV infusions and was approved in 2020 in the United States and European Union for the treatment of MM.^13,15^ The phase 1b PAVO study showed that DARA SC could be delivered with a reduced administration time (3–5 minutes), was well tolerated (only 16% of patients experienced IRRs), had acceptable pharmacokinetics (PK), and achieved deep responses in patients with RRMM.^16^ Similar results were observed in a small phase 1 study in Japanese patients.^17^ The phase 3 COLUMBA study demonstrated that DARA SC was noninferior to DARA IV in terms of efficacy and PK, with a similar safety profile and significantly reduced IRRs (DARA SC, 13% vs DARA IV, 34%; *P* < .0001).^18^

Studies in Chinese patients have shown DARA IV to be well tolerated and efficacious in both newly diagnosed MM and RRMM populations.^19–21^ The purpose of this phase 1 study was to evaluate safety, PK, and efficacy of DARA SC in Chinese patients with RRMM, as these results may impact the administration of DARA in this patient population.

## 2. MATERIALS AND METHODS

### 2.1. Study design and patients

MMY1010 (ClinicalTrials.gov Identifier: NCT04121260) is a multicenter, open-label, phase 1 study of Chinese patients with RRMM. The study and amendments were reviewed by an Independent Ethics Committee or Institutional Review Board at each site (Chinese Academy of Medical Sciences, XY2019031-EC-1/XY2019031-EC-2; Zhongda Hospital Southeast University, 2019ZDSYLL182-P01; Peking University Third Hospital, [2019] EC review number 126-01; Third Xiangya Hospital of Central South University, 19180, fast 20043; Nanfang Hospital of Southern Medical University, NFEC-2019-202). The study was conducted in accordance with the principles of the Declaration of Helsinki and consistent with Good Clinical Practice guidelines and applicable regulatory requirements. All patients provided written informed consent.

Eligible patients were aged ≥18 years with a documented diagnosis of MM (according to International Myeloma Working Group criteria),^22^ measurable serum or urine M-protein levels (or, for patients without measurable serum or urine M protein, measurable serum Ig free light chain and abnormal serum Ig κ/λ free light-chain ratio), and an Eastern Cooperative Oncology Group performance status score of 0 or 1. Patients with RRMM received ≥2 prior lines of therapy, including PI and IMiD, achieved a response (partial response or better) to ≥1 prior treatment regimen, had disease progression on or after the last therapy, and were naive to anti-CD38 therapy. Eligible patients had a hemoglobin level ≥7.5 g/dL, absolute neutrophil count ≥1.0 × 10^9^/L, platelet count ≥75 × 10^9^/L (patients in whom <50% of bone marrow nucleated cells were plasma cells; otherwise ≥50 × 10^9^/L), aspartate and alanine aminotransferase levels ≤2.5 ×upper limit of normal (ULN), total bilirubin ≤2.0 ×ULN (direct bilirubin ≤2.0 ×ULN in patients with congenital bilirubinemia), creatinine clearance ≥20 mL/min/1.73 m^2^, and corrected serum calcium ≤14 mg/dL or free ionized calcium ≤6.5 mg/dL.

Patients received a flat dose of 1800 mg DARA co-formulated with rHuPH20 (30,000 U in 15 mL) in a single, pre-mixed vial over 3 to 5 minutes by manual SC injection at alternating abdomen locations. Treatment cycle duration was 28 days. DARA SC was administered weekly for the first 8 weeks (cycles 1 and 2), every 2 weeks for 16 weeks (cycles 3–6), then every 4 weeks for cycle 7 and beyond until disease progression, unacceptable toxicity, or any other reason for discontinuation.

Patients received pre-dose and post-dose medications to reduce the incidence of IRRs. Prior to each study drug administration (within 1–3 hours), patients received acetaminophen (650–1000 mg IV or oral [PO]), diphenhydramine (25–50 mg IV or PO [or equivalent]), and methylprednisolone (100 mg IV or PO [or equivalent]; reduction to 60 mg after the second dose [in the absence of IRR adverse events {AEs} in the first 2 doses]). Pre-dose administration of a leukotriene inhibitor (montelukast 10 mg PO [or equivalent]) was optional on cycle 1 day 1 and could be administered up to 24 hours before administration as per investigator discretion. Patients also received post-dose oral methylprednisolone 20 mg (or equivalent) on the first 2 days following all DARA SC administrations. In the absence of IRR AEs after the first 3 administrations, post-dose corticosteroids were administered at the investigators’ discretion. Post-dose medications were administered for patients with a higher risk of respiratory complications (ie, those with mild asthma or patients with chronic obstructive pulmonary disease who have forced expiratory volume in 1 second <80%); these medications included diphenhydramine (or equivalent), leukotriene inhibitor (montelukast or equivalent), short-acting β_2_-adrenergic receptor agonist such as salbutamol aerosol, and control medications for lung disease (eg, inhaled corticosteroids ±long-acting β_2_-adrenergic receptor agonists for patients with asthma; long-acting bronchodilators such as tiotropium or salmeterol ±inhaled corticosteroids for patients with chronic obstructive pulmonary disease).

Each patient had a follow-up visit 8 weeks (±7 days) after final treatment dose. Follow-up continued until end of study, approximately 18 months after the last patient received the first dose of DARA SC.

### 2.2. Study endpoints and assessments

The primary endpoints of the study were PK parameters and safety of DARA SC. Secondary endpoints included overall response rate (ORR), duration of response, time to response, and the incidence of DARA and rHuPH20 antibodies.

The primary PK parameter was the observed concentration immediately prior to the next study drug administration (*C*_trough_) and was evaluated at the end of weekly dosing (cycle 3 day 1 pre-dose concentration). Maximum observed concentration (*C*_max_) was also assessed. Serial blood samples were collected pre-dose (day 1) and at 2 (day 1), 12 (day 1), 24 (day 2), 48 (day 3), and 72 hours (day 4) post-dose, followed by pre-dose samples on days 8, 15, and 22 of cycle 1; pre-dose on day 1 of cycle 2; pre-dose on day 1 and 72 hours (day 4) post-dose of cycle 3; and then pre-dose on day 1 of cycles 5, 7, and 8. Samples were also collected at the end of treatment and 8 weeks post-treatment.

Safety assessments included treatment-emergent AEs (TEAEs), clinical laboratory parameters, electrocardiograms, vital sign measurements, SC injection-site evaluations, and Eastern Cooperative Oncology Group performance status. TEAEs were assessed using the National Cancer Institute Common Terminology Criteria for Adverse Events version 4.03.

Blood samples were analyzed for anti-DARA and anti-rHuPH20 antibodies at pre-dose on cycle 1 day 1, cycle 1 day 15, cycle 5 day 1, and cycle 7 day 1, and at the end of treatment and 8 weeks post-treatment.

Tumor response was assessed in accordance with International Myeloma Working Group response criteria.^23^ Disease evaluations were performed by a central laboratory every 28 days (±7 days) until disease progression.

### 2.3. Statistical approach

The all-treated analysis set (all safety and efficacy analyses) included all patients who received ≥1 dose of DARA SC. The PK-evaluable analysis set included patients who received ≥1 dose of DARA SC and provided ≥1 post-dose PK sample. The primary PK parameter–evaluable analysis set included patients who received all 8 weekly DARA SC doses and provided a pre-dose PK sample on cycle 3 day 1. Noncompartment PK analysis was performed, and *C*_trough_ and *C*_max_ at each sample time point were summarized descriptively. The lower limit of quantification (LLOQ) was 0.20000 µg/mL. Other PK parameters included time to reach maximum observed serum concentration (*T*_max_) and area under the time curve from time 0 to 7 days post-dose (AUC_7days_). The immunogenicity analysis set included patients who received ≥1 dose of DARA SC and had appropriate samples for antibody detection.

No formal statistical hypothesis testing was conducted; all outcomes were summarized using descriptive statistics.

## 3. RESULTS

### 3.1. Patients and treatment

The first patient was enrolled on March 20, 2020. At the April 27, 2021, clinical cutoff, 21 patients were enrolled at 5 clinical sites in China and received ≥1 dose of DARA SC. The median age was 64 (range, 40–76) years, with 1 patient aged ≥75 years (**Table 1**). Most patients (12 patients; 57.1%) had standard cytogenetic risk; 8 (38.1%) patients had high-risk cytogenetic abnormalities, and the cytogenetic risk status of 1 (4.8%) patient was unknown. Patients received a median of 3 (range, 1–7) prior lines of therapy. More than half of patients were refractory to bortezomib (57.1%) or lenalidomide (66.7%), and nearly all (95.2%) were refractory to their last line of prior therapy. At the clinical cutoff, 10 (47.6%) patients discontinued treatment; reasons for discontinuation included progressive disease (n = 7), patient withdrawal (n = 2), and noncompliance (n = 1).

**Table 1:** Baseline demographic and clinical characteristics: all-treated analysis set.

Characteristic	DARA SC 1800 mg (N = 21)
Age	
Median (range), y	64 (40–76)
18–<65 y, n (%)	13 (61.9)
65–<75 y, n (%)	7 (33.3)
≥75 y, n (%)	1 (4.8)
Male, n (%)	7 (33.3)
Weight	
Median (range), kg ≤65 kg, n (%) >65 kg, n (%)	60 (46–87) 13 (61.9) 8 (38.1)
ECOG PS score, n (%)	
0	12 (57.1)
1	9 (42.9)
ISS stage,* n (%)	
I	9 (42.9)
II	7 (33.3)
III	5 (23.8)
Median (range) time from diagnosis, y	3.07 (1.2–7.6)
IgG myeloma, n (%)	14 (66.7)
Cytogenetic risk, n (%)†	
Standard risk	12 (57.1)
High risk	8 (38.1)
del17p	4 (19.0)
t(4;14)	4 (19.0)
t(14;16)	2 (9.5)
Unknown	1 (4.8)
Prior lines of therapy	
Median (range)	3 (1–7)
Number of prior lines of therapy, n (%)	
1	1 (4.8)
2	6 (28.6)
3	5 (23.8)
>3	9 (42.9)
Prior ASCT, n (%)	2 (9.5)
Prior PI, n (%)	21 (100)
Bortezomib	21 (100)
Prior IMiD, n (%)	21 (100)
Lenalidomide	19 (90.5)
Refractory to, n (%)	
Bortezomib	12 (57.1)
Lenalidomide	14 (66.7)
Both PI and IMiD	12 (57.1)
Last line of therapy	20 (95.2)

ASCT = autologous stem cell transplant, DARA SC = subcutaneous daratumumab, ECOG PS = Eastern Cooperative Oncology Group performance status, FISH = fluorescence in situ hybridization, IMiD = immunomodulatory drug, ISS = International Staging System, PI = proteasome inhibitor.

* ISS stage is derived based on the combination of serum β_2_-microglobulin and albumin.

† Assessed by FISH or karyotyping.

The median treatment duration was 6.5 (range, 1.4–10.2) months, and the median number of treatment cycles received was 8 (range, 2–12). The median relative dose intensity was 100% (range, 87.5%–100%). The median duration of the first, second, and all subsequent injections was 4.0 (range, 3.0–5.0), 3.0 (3.0–5.0), and 3.0 (2.0–5.0) minutes, respectively.

### 3.2. Pharmacokinetics

**Figure 1.**
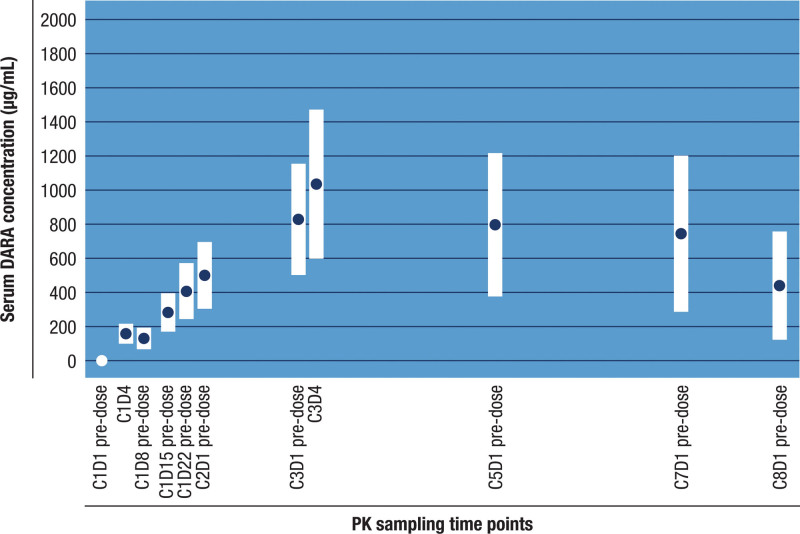
Mean and SDs of serum DARA *C*_max_ and *C*_trough_ (μg/mL): PK-evaluable analysis set. At each time point, concentration values are plotted on a linear scale and the error bars are mean ± SD. Concentration values <LLOQ are considered as 0. Samples with a collection time out of defined windows and samples collected after an incomplete dose (<80% of the intended dose was administered) and prior to the next complete dose were not included. C = cycle, *C*_max_ = maximum observed concentration, *C*_trough_ = trough (pre-dose) concentration observed immediately prior to the next study drug administration, D = day, DARA = daratumumab, LLOQ = lower limit of quantification, PK = pharmacokinetic, SD = standard deviation.

The PK-evaluable analysis set included all 21 patients, with 18 patients in the primary PK parameter–evaluable analysis set. Detectable DARA concentrations were observed at 2 hours post-first dose in most patients, with mean serum concentrations peaking at 3 days post-dose (cycle 1 day 4) and sustaining until the end of the 1-week dosing interval. DARA accumulation continued throughout weekly dosing and decreased slightly starting at the every-2-week dosing period and subsequent every-4-week dosing period. On day 4 after the last weekly dose (cycle 3 day 4), the mean (standard deviation [SD]) *C*_max_ was 1036 (437) µg/mL, which was 6.5-fold greater than peak concentration (cycle 1 day 4, 159 [58.6] µg/mL). Mean (SD) *C*_trough_ concentrations increased from 131 (63.6) µg/mL following the first dose (cycle 1 day 8 pre-dose) to 829 (326) µg/mL after the eighth dose at end of the weekly dosing schedule (cycle 3 day 1 pre-dose). Subsequently, mean (SD) *C*_trough_ decreased to 745 (458) µg/mL at end of the every-2-week dosing schedule (cycle 7 day 1 pre-dose) and 440 (318) µg/mL after 1 cycle on the every-4-week dosing schedule (cycle 8 day 1 pre-dose; **Fig. 1**).

Following the first drug administration, the mean (SD) *C*_max_ and AUC_7days_ were 163 (57.4) μg/mL and 21,354 (8648) h·μg/mL, respectively. Median *T*_max_ after the first drug administration was 70.8 hours, while individual *T*_max_ values ranged from 46.1 to 173.9 hours. The mean (SD) maximum *C*_trough_ (cycle 3 day 1 pre-dose) based on the primary PK parameter–evaluable analysis set was 826 (335) µg/mL (**Table 2**). Further, mean (SD) maximum *C*_trough_ in the lower–body weight subgroup (≤65 kg) was numerically higher than that in the higher–body weight subgroup (>65 kg).

**Table 2:** Maximum *C*_trough_ for end of weekly dosing: primary PK parameter–evaluable analysis set.

Maximum *C*_trough_ on cycle 3 day 1, μg/mL	DARA SC 1800 mg		
	Total (N = 18)	≤65 kg (n = 12)	>65 kg (n = 6)
Mean (SD)	826 (335)	964 (295)	549 (227)
Geometric mean	755	917	512
Median	847	918	509
Range	(288–1419)	(458–1419)	(288–927)
CV, %	40.5	30.6	41.3

*C*_trough_ = observed concentration immediately prior to the next study drug administration, CV = coefficient of variation, DARA SC = subcutaneous daratumumab, PK = pharmacokinetic, SD = standard deviation.

### 3.3. Safety

The most frequently reported (≥20%) TEAEs with DARA SC were anemia (52.4%), leukopenia (52.4%), neutropenia (47.6%), thrombocytopenia (47.6%), lymphopenia (42.9%), and hyperglycemia (28.6%; **Table 3**). The most common (≥10%) grade 3 or 4 TEAEs were lymphopenia (33.3%), anemia (28.6%), thrombocytopenia (23.8%), leukopenia (19.0%), neutropenia (14.3%), and pneumonia (14.3%). Grade 3 or 4 infections occurred in 4 (19.0%) patients: 3 (14.3%) with pneumonia and 1 (4.8%) with herpes zoster. Seven (33.3%) patients had serious TEAEs, 6 of which were considered drug-related. Serious TEAEs that occurred in >1 patient were pneumonia and thrombocytopenia (n = 2 each). The percentages of patients who experienced grade 3 or 4 and serious TEAEs were comparable between the lower–body weight subgroup (≤65 kg) and the higher–body weight subgroup (>65 kg); in these subgroups, grade 3 or 4 TEAEs occurred in 69.2% and 62.5% of patients, respectively, and serious TEAEs occurred in 30.8% and 37.5% of patients, respectively. No treatment discontinuations or deaths (within 30 days of the last dose or within 60 days of the first dose) due to TEAEs were observed. Three (14.3%) patients died during study: 1 due to a serious AE of gastrointestinal hemorrhage that occurred beyond 30 days of the last dose (and was not considered to be a TEAE) and 2 patients due to progressive disease. One (4.8%) patient experienced a treatment-emergent IRR, which was grade 1 and occurred after the first administration of DARA SC. Two (9.5%) patients had treatment-emergent injection-site reactions (grade 1 injection-site swelling and grade 1 erythema; n = 1 each). No second primary malignancy, hepatitis B reactivation, or COVID-19 infection events were reported.

**Table 3:** TEAEs: all-treated analysis set.

TEAE, n (%)	DARA SC 1800 mg (N = 21)	
	Any grade >10%	Grade 3 or 4 >1 patient
Hematologic		
Anemia	11 (52.4)	6 (28.6)
Leukopenia	11 (52.4)	4 (19.0)
Neutropenia	10 (47.6)	3 (14.3)
Thrombocytopenia	10 (47.6)	5 (23.8)
Lymphopenia	9 (42.9)	7 (33.3)
Nonhematologic		
Hyperglycemia	6 (28.6)	1 (4.8)
Pneumonia	4 (19.0)	3 (14.3)
Herpes zoster	4 (19.0)	1 (4.8)
Hyperuricemia	4 (19.0)	0
Hypokalemia	4 (19.0)	0
Back pain	4 (19.0)	0
Constipation	4 (19.0)	0
Pyrexia	3 (14.3)	0
Blood pressure increased	3 (14.3)	0
Tachycardia	3 (14.3)	0
Nasopharyngitis	3 (14.3)	0
Upper respiratory tract infection	3 (14.3)	0

DARA SC, subcutaneous daratumumab, TEAE = treatment-emergent adverse event.

### 3.4. Immunogenicity

All 21 patients were included in the immunogenicity-evaluable analysis population. No patient tested positive for anti-DARA or anti-rHuPH20 antibodies.

### 3.5. Efficacy

**Figure 2.**
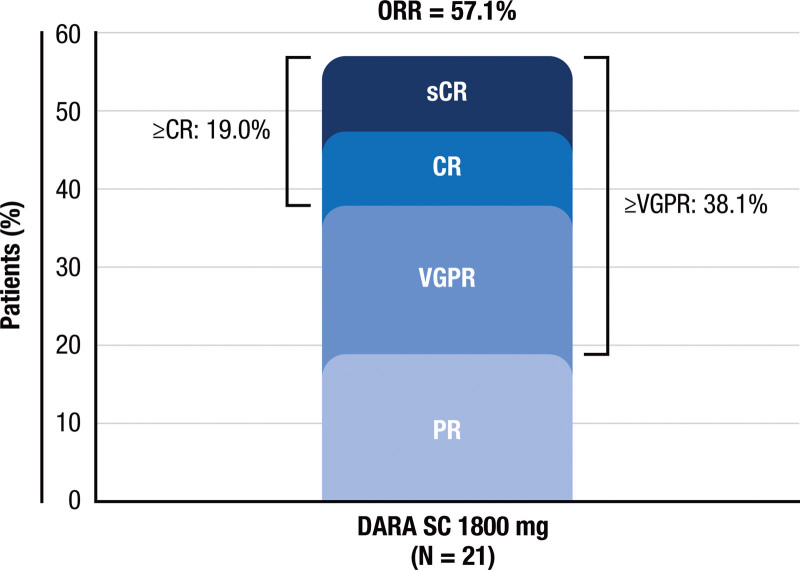
Summary of responses to DARA SC 1800 mg. Responses were evaluated in the all-treated population, which included all patients who received ≥1 dose of the study drug. CR = complete response, DARA SC, subcutaneous daratumumab, ORR = overall response rate, PR = partial response, sCR = stringent complete response, VGPR = very good partial response.

At a median follow-up of 7.5 months, the ORR with DARA SC was 57.1%, with a very good partial response or better rate (≥VGPR) of 38.1% and a complete response or better rate (≥CR) of 19.0% (**Fig. 2**). In the lower–body weight subgroup (≤65 kg), the ORR was 69.2%, ≥VGPR was 46.2%, and ≥CR was 23.1%. In the higher–body weight subgroup (>65 kg), the ORR was 37.5%, ≥VGPR was 25.0%, and ≥CR was 12.5%. The median time to first response was 0.95 (range, 0.95–8.34) months, median time to best response was 3.07 (range, 0.95–8.34) months, and median duration of response was not reached. At the clinical cutoff, median progression-free survival (PFS) was not reached; the estimated 9-month PFS rate was 61.4%. OS data are not mature; 3 (14.3%) patients died by the data cutoff, with an estimated 9-month OS rate of 78.8%.

## 4. DISCUSSION

MMY1010 is the first clinical study to evaluate the PK and safety of DARA SC in Chinese patients with MM. Results from the phase 1b PAVO study indicated that flat dosing of DARA SC (1800 mg DARA co-formulated with rHuPH20) was a feasible approach for the treatment of RRMM, conferring benefits of reduced administration time and decreased IRR rates compared to DARA IV.^16^ Similar to PAVO, in the current study, Chinese patients with heavily pre-treated RRMM received DARA SC monotherapy until disease progression or death. Almost half (42.9%) of the patients had received >3 prior lines of therapy, more than half (57.1%) were refractory to both prior PI and IMiD treatment, and nearly all (95.2%) were refractory to their last line of prior therapy. The median duration of DARA SC injections was 3 to 4 minutes, a clear time advantage over IV administration, which had a median duration of 7, 4, and 3 hours for the first, second, and subsequent infusions, respectively, in clinical studies.^14^

The mean (SD) maximum *C*_trough_ (cycle 3 day 1 pre-dose) was 826 (335) μg/mL, which was within a similar exposure range compared with a previous global phase 3 DARA SC study (COLUMBA),^18^ a Western population–based phase 1b DARA SC study (PAVO),^16^ and 2 Asian population–specific phase 1 studies (MMY1008 [Japanese patients; DARA SC]^17^ and MMY1003 [Chinese patients; DARA IV]^21^). As expected for a monoclonal antibody administered SC by flat dose,^24^ higher exposures were observed in patients of lower body weight and vice versa; however, these results should be interpreted with caution due to small sample sizes. Taken together, these PK results suggest that flat-dose administration of DARA SC provides adequate exposure in Chinese patients with RRMM.

The safety profile of DARA SC monotherapy in Chinese patients with RRMM was consistent with the safety profiles of DARA IV and DARA SC observed in other local and global studies. No new safety concerns were identified. The safety profile of DARA SC was consistent between body weight subgroups; of note, there was no substantial increase in TEAEs in patients with lower body weights (≤65 kg) compared to higher body weights (>65 kg). At the clinical cutoff, no patient had TEAEs leading to treatment discontinuation, and no deaths were due to TEAEs. The observed TEAE frequencies in this study were higher than those observed in the DARA SC group in the global COLUMBA study.^18^ However, similar patterns were found in hematologic and chemical laboratory abnormalities during treatment between COLUMBA and MMY1010; thus, the higher reporting frequencies of TEAEs overall in MMY1010 may have been due to differences in investigators’ clinical discretion in reporting laboratory abnormalities as TEAEs. Observed TEAE frequencies in this study with DARA SC were similar to those reported in Chinese patients with RRMM receiving DARA IV at 16 mg/kg in a phase 1 study (MMY1003).^21^ For instance, comparable rates of grade 3/4 anemia (28.6% in MMY1010; 29.8% in MMY1003), leukopenia (19.0%; 23.4%), and neutropenia (14.3%; 19.1%) were observed. Conversely, in a real-world study of DARA IV use in Korean patients with RRMM, rates of grade 3/4 anemia and neutropenia were higher with DARA IV (MMY1010, 28.6% and 14.3%, respectively; real-world, 37.5% and 25.0%), whereas rates of grade 3/4 thrombocytopenia were lower (MMY1010, 23.8%; real-world, 6.3%).^25^ Incidences of IRRs and injection-site reactions were low during this study (1 and 2 patients, respectively; all grade 1) and were consistent with those seen in the DARA SC group of the COLUMBA study (IRRs, 4.8% in MMY1010 and 12.7% in the COLUMBA DARA SC group; injection-site reactions, 9.5% and 6.9%, respectively).^18^ For comparison, 27.7% of Chinese patients in the MMY1003 study who were treated with DARA IV at a dose of 16 mg/kg experienced IRRs, which is much higher than that observed in the current study with DARA SC (4.8%).^21^ A higher rate (42.9%) of IRRs following DARA IV was also reported in a real-world retrospective study of heavily pre-treated Asian patients with RRMM.^26^ In addition to low incidences of IRRs and injection-site reactions, there was a low incidence of anti-DARA or anti-rHuPH20 antibodies (0% for both) in the current study, indicating a low risk of DARA immunogenicity when administered SC. The rate of anti-DARA antibodies was comparable to that observed in the COLUMBA study, in which 0% and <1% of patients receiving DARA SC or DARA IV, respectively, presented with anti-DARA antibodies.^18^ Despite the small sample size (n = 21), the overall safety profile demonstrated in this study was consistent with the known safety profile of DARA SC monotherapy and DARA IV (except for a reduction in IRRs).

Data from this study showed favorable clinical efficacy of DARA SC monotherapy in Chinese patients with RRMM. In this heavily pre-treated treatment-refractory population, DARA SC demonstrated remarkable clinical activity, achieving an ORR of 57.1%. These efficacy data are consistent with local and global monotherapy studies for DARA; ORRs were 42.6% with DARA IV 16 mg/kg in the MMY1003 study in Chinese patients and 41.1% with DARA SC in the global COLUMBA study.^18,21^ In this study, ORR, ≥VGPR, and ≥CR were higher in patients of lower body weight than those of higher body weight. Note that small sample sizes may limit the above comparisons, and results should be interpreted with caution.

## 5. CONCLUSIONS

In summary, the results of this phase 1 study of DARA SC monotherapy in Chinese patients with RRMM are consistent with known safety profiles of DARA IV and DARA SC, with no new safety concerns. Administration time was dramatically reduced relative to IV administration, and the rates of IRRs were markedly lower. PK characteristics of DARA SC observed in Chinese patients were consistent with previous observations. Despite this study’s small sample size, DARA SC demonstrated robust clinical activity, with an ORR of 57.1% in a heavily pre-treated population. Collectively, the data from MMY1010 demonstrated a favorable benefit/risk profile of DARA SC monotherapy for the treatment of Chinese patients with RRMM.

## ACKNOWLEDGMENTS

This study was sponsored by Janssen Research & Development, LLC.

We thank the patients who participated in this study, their families, and the staff members at the trial sites who cared for them; the members of the data and safety monitoring committee; and the representatives of the sponsor, who were involved in data collection and analyses. Medical writing and editorial support were provided by Deborah Bouis, PhD, of Lumanity Communications Inc., and were funded by Janssen Global Services, LLC.

## AUTHOR CONTRIBUTIONS

G.A.: Data curation, investigation, writing-review & editing; Z.G.: Data curation, investigation, writing-review & editing; H.J.: Data curation, investigation, writing-review & editing; J.L.: Data curation, investigation, writing-review & editing; G.Y.: Data curation, investigation, writing-review & editing; R.F.: Data curation, investigation, writing-review & editing; Z.X.: Data curation, investigation, writing-review & editing; M.Q.: Data curation, investigation, writing-review & editing; J.W.: Data curation, investigation, writing-review & editing; J.S.: Data curation, investigation, writing-review & editing; W.Z.: Data curation, investigation, writing-review & editing; B.S.: Writing-review & editing; D.Z.: Writing-review & editing; X.C.: Writing-review & editing; C.C.: Writing-review & editing; L.Q.: Conceptualization, investigation, resources, supervision, writing-review & editing.
